# Supplementary material for: The interactome of intact mitochondria by cross-linking mass spectrometry provides evidence for coexisting respiratory supercomplexes
Source: Mol Cell Proteomics. 2017 Dec 8;17(2):216–32. doi: 10.1074/mcp.RA117.000470 (PMC5795388; doi:10.1074/mcp.RA117.000470)
Supplement: Supplemental Data [file supp_RA117.000470_133922_0_supp_23978_fzffwf.zip › spectra_annotation/mito_spectra_annotation/150-1-8-1-27-1.pdf]

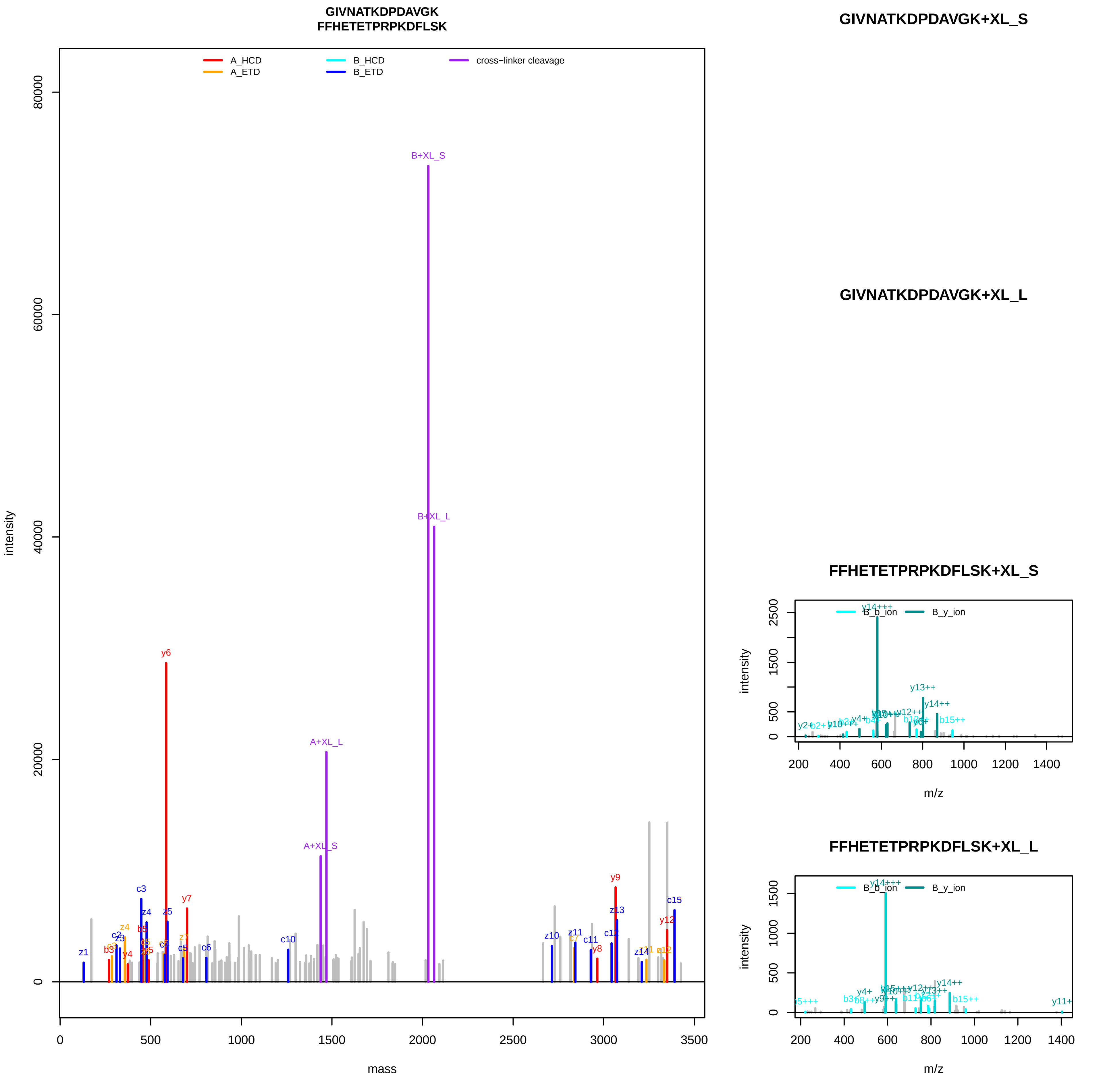
GIVNATKDPDAVGK
FFHETETPRPKDFLSK
A_HCD
A_ETD
B_HCD
B_ETD
cross−linker cleavage
B+XL_S
B+XL_L
A+XL_L
A+XL_S
y6
y7
y9
y12
c3
z4
z5
c15
z13
c10
z10
z11
c11
c6
z1
y8
z14
intensity
mass
0
20000
40000
60000
80000
0
500
1000
1500
2000
2500
3000
3500
GIVNATKDPDAVGK+XL_S
GIVNATKDPDAVGK+XL_L
FFHETETPRPKDFLSK+XL_S
B_b_ion
B_y_ion
y14+++
y13++
y14++
b15++
intensity
m/z
0
500
1500
2500
200
400
600
800
1000
1200
1400
FFHETETPRPKDFLSK+XL_L
B_b_ion
B_y_ion
y14+++
y14++
b15++
y11+
intensity
m/z
0
500
1000
1500
200
400
600
800
1000
1200
1400
